# Reward-Related Suppression of Neural Activity in Macaque Visual Area V4

**DOI:** 10.1093/cercor/bhaa079

**Published:** 2020-04-30

**Authors:** Katharine A Shapcott, Joscha T Schmiedt, Kleopatra Kouroupaki, Ricardo Kienitz, Andreea Lazar, Wolf Singer, Michael C Schmid

**Affiliations:** 1 Schmid Lab, Ernst Strüngmann Institute (ESI) for Neuroscience in Cooperation with Max Planck Society, Frankfurt a. M. 60528, Germany; 2 Singer Lab, Ernst Strüngmann Institute (ESI) for Neuroscience in Cooperation with Max Planck Society, Frankfurt a. M. 60528, Germany; 3 Singer Group, Frankfurt Institute for Advanced Studies, Frankfurt a. M. 60438, Germany; 4 Biosciences Institute, Faculty of Medical Sciences, Newcastle upon Tyne NE2 4HH, UK; 5 Epilepsy Center Frankfurt Rhine-Main, Center of Neurology and Neurosurgery, Goethe University, Frankfurt a. M. 60528, Germany; 6 Faculty of Science and Medicine, University of Fribourg, Fribourg 1700, Switzerland

**Keywords:** attention, electrophysiology, normalization, reward, visual cortex

## Abstract

In order for organisms to survive, they need to detect rewarding stimuli, for example, food or a mate, in a complex environment with many competing stimuli. These rewarding stimuli should be detected even if they are nonsalient or irrelevant to the current goal. The value-driven theory of attentional selection proposes that this detection takes place through reward-associated stimuli automatically engaging attentional mechanisms. But how this is achieved in the brain is not very well understood. Here, we investigate the effect of differential reward on the multiunit activity in visual area V4 of monkeys performing a perceptual judgment task. Surprisingly, instead of finding reward-related increases in neural responses to the perceptual target, we observed a large suppression at the onset of the reward indicating cues. Therefore, while previous research showed that reward increases neural activity, here we report a decrease. More suppression was caused by cues associated with higher reward than with lower reward, although neither cue was informative about the perceptually correct choice. This finding of reward-associated neural suppression further highlights normalization as a general cortical mechanism and is consistent with predictions of the value-driven attention theory.

## Introduction

The reward system of the brain is essential for the survival of complex organisms because it generates the teaching signal that encourages behaviors contributing to selective fitness (Dayan and Balleine 2002; Wise 2004). Conversely, in humans, failures in reward management can be the cause of addiction, which adversely affects the health of millions of people worldwide (Hyman et al. 2006). It is therefore crucial to gain a better understanding of how reward signals impact the brain’s responses to stimuli and influence behavior.

To understand better how reward influences behavior, we investigated how nonsensory aspects of a task, expected reward and behavioral choice, interact with the sensory representation of stimuli in the early visual cortex. To this end we adopted a two-alternative forced-choice (2AFC) reward maximization task previously used for a study in a higher visual area (Rorie et al. 2010) and analyzed responses of neurons in monkey V4 to visual stimuli under multiple reward conditions. Monkeys had to perform a motion discrimination task (upward or downward) and indicate the perceived motion with a saccade in the direction of motion toward one of two targets, located above and below the stimulus. The color of these targets could change and indicated to the monkey the amount of reward associated with the correct choice in the respective directions. It has been shown previously that allocation of attention to a stimulus enhances responses in V4 (Maunsell 2004). Assuming that more attention is allocated if stimuli are associated with high rather than low reward, we predicted that the responses to the motion stimulus would increase when the upcoming trial was associated with high compared to low reward. Unexpectedly, we observed the opposite effect. We reasoned that this counterintuitive outcome can be accounted for if the cues predicting reward value had themselves attracted attention, the amount of allocated attention depending on the amount of predicted reward.

Such value-driven allocation of attention has been proposed in the “value-driven theory of attentional selection” (Anderson 2013, 2016). This theory posits that stimuli previously associated with reward gain increased attentional priority. Most theories on the control of attention distinguish between bottom-up and top-down allocation. Salient stimuli attract attention and thereby get processed preferentially (bottom-up allocation). Likewise, attention can be actively allocated to goal- or task-relevant stimuli independently of their salience (top-down allocation). Both mechanisms of attention allocation are considered by the value-driven mechanism of attentional selection as helpful to maximize reward. More salient stimuli are likely to be behaviorally relevant, and top-down goals are often established in order to gain reward. In addition, the value-driven attention theory makes the unique prediction that attention will still be drawn to reward-associated stimuli even when this attentional capture is behaviorally disadvantageous. No other attentional theory predicts that previously rewarded stimuli that are neither salient nor part of a current goal should attract attention. However, this prediction is in agreement with a growing body of evidence in human psychophysics studies, which document exactly this type of disadvantageous attentional capture of nonsalient rewarded stimuli (Libera and Chelazzi 2009; Hickey and Van Zoest 2012; Failing and Theeuwes 2017; Le Pelley et al. 2017; Marchner and Preuschhof 2018). This unconscious attentional capture can take place even after delays of several months from the original reward association event (Anderson and Yantis 2013). In addition, human neuroimaging studies have found increases of neural activity in response to previously rewarded but task-irrelevant stimuli in both the extrastriate visual cortex and striatum (Anderson et al. 2014; Donohue et al. 2016; Anderson 2017; for review see Anderson 2019).

Further support for the value-driven attention theory may come from electrophysiological recordings of neural activity. A common finding across visual and parietal cortical brain areas is that reward and attention cause comparable changes in neural responses (Peck et al. 2009; Stanisor et al. 2013). However, in area V4, a midlevel visual area subject to intense attention research (Roe et al. 2012), a study by Baruni et al. (2015) concluded that reward and attention had differential effects. They found that only the reward associated with the stimulus inside the V4 receptive fields (RF), but not the attentional allocation, influenced the neurons’ responses. The authors discuss that this result may have been caused by their task failing to recruit normalization mechanisms. The “normalization mechanism” of attention posits that changes in neural responses to attended stimuli are determined by attention interacting with divisive normalization mechanisms, which modulate both excitatory and inhibitory influences on the neuron’s responses (Lee and Maunsell 2009; Reynolds and Heeger 2009; Sundberg et al. 2009). This model accounts for complex attention-related effects and explains the finding that, in the presence of multiple stimuli, attention allocated to a stimulus in the inhibitory surround of a neuron’s RF suppresses responses to a center stimulus (Sundberg et al. 2009). We hypothesized that combining the value-driven attention theory with the normalization mechanism of attention could account for our findings. In the Baruni et al. study, the spatial layout of the two stimuli was such that the effect of surround suppression was weak. Thus, attention-dependent normalization mechanisms may not have been engaged. Therefore, it remains an open question whether or not in visual area V4 “attentional” normalization mechanisms are engaged by stimuli associated with reward. Our results suggest that in a 2AFC task, reward cues do engage normalization mechanisms, which provides evidence for the value-driven attention theory in macaque visual area V4.

## Materials and Methods

### Subjects and Surgical Procedures

Two male rhesus macaques (*Macaca mulatta*) participated in the study. All procedures were in accordance with the animal welfare guidelines of the Regional Board of Darmstadt (F149/05) and the European Union’s Directive 2010/63/EU. Animals received controlled access to fluids during experimental periods to ensure motivation for the cognitive experiments in accordance with regulations.

For each surgery anesthesia was induced by injection and was then maintained using gas. In an initial surgery, custom-fitted titanium head immobilization posts were implanted onto the front of the skull (Lanz et al. 2013), and in a later surgery, two Utah arrays (Blackrock Microsystems) were inserted subdurally in areas V1 and V4 in each monkey. Only results from the area V4 arrays are reported here. A reference wire was placed under the dura toward the cerebellum and parietal cortex in both monkeys. Throughout the study veterinarians and technicians as well as scientists monitored animal welfare.

### Behavioral Setup

The monkeys were placed in a dark and electrically isolated booth and then headfixed before each recording session. They were positioned ∼80 cm from a 22″ LCD Samsung 2233RZ monitor of 1680 × 1050 resolution running at 120 Hz. This monitor was chosen due to its suitability for vision research and precise timing (Wang and Nikolic 2011). Most stimuli were presented on the monitor using the MonkeyLogic toolbox (Asaad and Eskandar 2008) with MATLAB 2011 (The Mathworks Inc.). The RF mapping stimuli were presented using Psychtoolbox (Pelli 1997). Grating stimuli were square-wave gratings of 1.5–3° (visual degrees) with a spatial frequency of 1°.

Eye movements and pupil diameter of one eye were tracked using the infrared camera system EyeLink 2000 (SR Research Ltd) at 500 Hz. The eye movements of the monkey were calibrated on each day before any sessions were run.

### Behavioral Task

**Figure 1:**
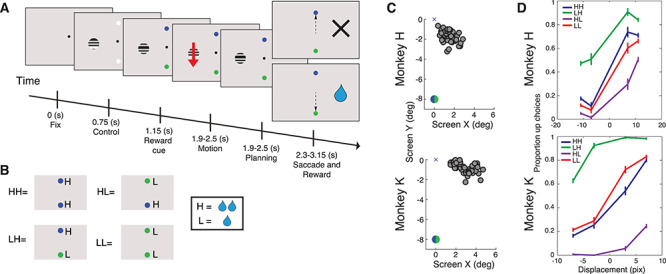
Reward biases behavioral choice during perceptual judgment task. (*A*) Layout of the 2AFC variable reward task. The single grating was positioned near the center of the RF across all array electrodes. (*B*) The reward conditions. The first letter signifies the reward value of the lower cue, and the second letter signifies the reward value of the top cue. Each drop signifies a single pulse of reward given to the monkey for a correct saccade to that target. In this example blue signified high reward to the monkey, but this was switched in the second week of recordings so that in the analyzed data, both blue and green can signify both high and low reward. (*C*) Location of the centers of RFs (see Methods) for all electrodes. The upper cross corresponds to the central fixation spot, and the lower circle illustrates the location of the lower reward cue. See Supplementary Fig. 2 for details of stimulus positions and RF sizes on the screen. (*D*) Proportion of saccades to upper target. Colors indicate reward conditions. Monkeys were more biased for difficult trials (small motion displacements) than easy trials (large motion displacements). Both monkeys H and K were biased toward the lower target although this was much more pronounced in monkey H. Error bars indicate SEM.

The monkeys performed a two-alternative forced-choice motion discrimination task (see Fig. 1*A*). Monkeys had to maintain their eye position within a 0.8–1° radius of a white fixation point on a gray background. After a baseline period of 750 ms (for timings see Fig. 1*A*; for locations see Supplementary Fig. 2), a static grating (monkey H: contrast 100%, radius 1°, spatial frequency 1 cycles/°; monkey K: contrast 100%, radius 1.25°, spatial frequency 1 cycles/°) and two white saccade targets (radius 0.4°) appeared as the stimulus baseline period (400 ms). Then the amount of reward was cued, as described further below, by changing the colors of the saccade targets (to blue or green, luminance was not matched). After a variable delay of 750–1350 ms, the grating then displaced between one frame and the next, perceptually a small apparent motion event, which the monkey needed to discriminate the direction of. The size of the displacement was between 3 and 11 pixels. After a variable delay of 400–650 ms, the fixation point disappeared, and a saccade was performed to one of the saccade targets. The monkey received a juice reward if it saccaded in the same direction as the motion event.

The reward value of a correct saccade, either one or two pulses of juice, was denoted by the color of the saccade target (either green or blue). The duration of each pulse was specific for each subject, but two pulses always produced twice the reward of a single pulse, keeping the relative amount of reward constant. One of the two colors would indicate high (H) reward and the other low (L) reward. This resulted in four conditions: HH, HL, LH, and LL (see Fig. 1*B*). The first letter corresponds to the value of a correct downward saccade, while the second is the value of a correct upward saccade. We switched the meaning of the cue color once (from blue designating high reward to low reward and vice-versa for green) and used 100 trials to retrain reward value. We ensured that during these trials, the subjects had become biased toward the new high reward color before continuing with the measurements. These training trials were excluded from all analyses.

### Neurophysiological Recordings

Recordings took place in an electrically isolated booth. The Utah array electrodes were arranged in an 8 × 8 grid with 0.4-mm spacing, four rows had electrode lengths of 0.6 mm, and four rows had electrode lengths of 1 mm. Due to the curved shape of the cortex, it is unlikely that these two electrode lengths were in separate layers. The electrode impedances were between 70 and 800 kΩ at 1000 kHz. The signals were amplified, digitized at 30 kHz directly at the connector, transferred via optic fiber, and recorded (Blackrock Microsystems).

### Data Analysis

All data were analyzed with the MATLAB (2013) or 2018, MathWorks) toolbox FieldTrip (Oostenveld et al. 2011) and custom-written analysis scripts. We recorded 10 sessions in each monkey. The number of trials from green or blue sessions was balanced by randomly removed trials. For the analysis of suppression, we included all other completed trials, but for the analysis of motion, trials were excluded with smaller motion displacement and incorrect trials to control for inattention.

### Pupil and Eye Movement Analysis

Pupil data were normalized to a percentage of the mean pupil diameter of each session, while the eye was within the fixation window. For each trial the absolute change from the mean baseline of −0.8 to 0.2 s relative to cue onset was calculated. Due to the slow nature of pupil responses (Beatty and Lucero-Wagoner 2000), pupil data were averaged from 0.2 s after cue onset until the response.

Microsaccades (MS) were detected by Engbert and Kliegl (2003) as events with a velocity of over five standard deviations from the median on each trial and lasting for a minimum of three samples (15 ms for our eye data sampling rate of 500 Hz). The rate in a window of 0.1–0.3 s postcue was used for the statistical analysis with a two-sample chi-squared test.

### Estimating MUA Activity

To obtain an estimate of multiunit activity (MUA) (Legatt et al. 1980; Supèr and Roelfsema 2005), the raw signal was high-pass filtered at 300 Hz with an eighth-order zero-phase Chebyshev filter, rectified, low-pass filtered at 120 Hz with a sixth-order zero-phase Butterworth filter, and downsampled to 500 Hz, yielding a quasi-continuous measure of high-frequency field power.

### RF Mapping

In order to estimate RF locations, a bar mapping method was used as by Fiorani et al. (2014). A white bar of width 0.2° of visual angle was swept over a gray background at a speed of 10°/s with eight evenly spaced orientations. The RF location was estimated from the neural responses to each orientation using the center of a 2D Gaussian fit to the backprojected RF map for each electrode with an estimated response delay (see Fiorani et al. 2014 for full details). This mapping procedure was performed once before the start of the experiment.

### MUA Analysis

To baseline the MUA, the mean activity in the time period from 0.3 to 0.5 s poststimulus was determined for each electrode for a whole session, and then the percent change from this stimulus baseline was calculated. To quantify neural suppression, on each trial the average stimulus baseline activity of each electrode was subtracted from the average of the cue-related activity (0.5–0.7 s poststimulus onset). To calculate the late suppression effect, a window of 1–1.4 s poststimulus onset was used; trials in which there was a stimulus motion event during this period were excluded. To calculate the MUA motion response on each trial, the average activity in the period from 0.05 to 0.2 s postmotion onset was used.

### Classification

MUA data was classified into different classes per trial (e.g., reward condition) using a linear naive Bayes classifier (MATLAB 2013) trained on average MUA activity in 100-ms time bins at 10-ms intervals. Classification performance was assessed using 10-fold validation. Trials were first balanced per class and per reward color scheme by randomly subselecting an equal number of trials.

### Statistics

To test for significance differences between conditions, Wilcoxon rank sum (WRS) tests were performed on the median of trials pooled over all sessions across electrodes. Plots showing mean array activity were smoothed with a Gaussian kernel for display purposes. Error bars on all plots are standard error of the mean (SEM) across trials.

To compare the classifier performance between conditions, unpaired *t*-tests were used. Each time bin was tested in this way, and the calculated *P* value was corrected for multiple comparisons using Holm–Bonferroni correction.

A regression was performed on the median of the suppression for each electrode against the distance in visual degrees of the electrode RF from the reward cue.

A linear model was built (fitlm, MATLAB 2018) to predict the suppression per trial based on the reward condition, cue color, channel label, session number, and the interaction between reward and color. For monkey K only the stimulus position was also used as a factor.

## Results

To investigate the effect of reward on area V4, we trained monkeys to perform a 2AFC motion discrimination task in which the amount of reward for a successful discrimination was varied independently of the motion direction (see Fig. 1*A* and Rorie et al. 2010). The task was initially identical for all conditions. Monkeys were asked to first fixate on a fixation spot, and then a grating stimulus and two white saccade targets appeared. The grating was positioned near the centers of the RFs of all recording sites covered by the electrode array (see Methods, Fig. 1*C*, Supplementary Figs. 2 and 6), while the saccade targets were positioned 8° (visual degrees) above and below the fixation point. After this stimulus baseline, the two saccade targets changed color. They assumed the additional function of cues which indicated the reward value of correct saccades to each location (one drop of juice [low reward; L] or two drops [high reward; H], see Fig. 1*B*). This resulted in four possible conditions, HH and LL, in which both saccade directions led to high or low reward, respectively, and HL and LH, in which the two saccade targets were associated with different reward magnitudes. For conditions HL and LH, the first letter indicates the reward value of the lower cue and the second that of the upper cue (see Fig. 1*B*). Following a variable delay after presentation of the grating and the saccade targets, the grating moved by a few pixels either downward or upward (see Methods). After another delay the monkey was allowed to saccade to one of the cues and was rewarded only if it chose the cue in the direction corresponding to the grating movement.

### Reward Contingencies Affect Behavior

We first examined the behavior of the monkeys separately for each reward condition to confirm that our results align with those found previously on similar reward varying tasks: Macaques are able to maximize the total amount of reward they receive when performing discrimination tasks with differing reward amounts (Feng et al. 2009; Rorie et al. 2010), and they do so by biasing their choices to the highly rewarded option in an optimal manner. We found that for conditions HH and LL, in which downward and upward directions were equally rewarded, both monkeys made a similar percentage of upward responses as expected (see Fig. 1*D*). When there was unequal reward available (conditions HL and LH), the monkeys showed a bias toward the higher rewarded direction (see Fig. 1*D*, Supplementary Fig. 1*A*), as demonstrated previously (Feng et al. 2009; Rorie et al. 2010). In the HL condition (higher reward was given for correct responses to the downward motion), there were 40.0% and 72.6% more downward responses than in the LH condition (higher reward for upward motion) for monkeys H and K, respectively. A binomial test showed that both monkeys were significantly more biased toward the higher rewarded target than would be expected by chance for condition LH *P* < 0.001 (*n* = 2102) and *P* < 0.001 (*n* = 3058) for monkey H and monkey K, respectively, for condition HL *P* < 0.001 (*n* = 2349) and *P* < 0.001 (*n* = 3231). We additionally found that the monkeys’ responses were more biased toward targets predicting high reward in difficult trials (small motion displacements) than easy trials (large motion displacements). For monkeys H and K, 8.65% and 12.22% of the responses were biased toward high reward for the difficult trials as demonstrated previously (Feng et al. 2009; Rorie et al. 2010). These findings indicate that both monkeys understood the task and attended to the motion direction and had additionally learnt how to optimize total reward.

We additionally found other behavioral evidence that the monkeys were affected by the reward contingencies of our task. As expected from previous work, monkeys were even more biased to the highly reward direction with increased task difficulty, another strategy to maximize reward (see Fig. 1*D*; two proportion *z*-test; *P* < 0.001 and *P* < 0.001 for monkeys H and K, respectively). Additionally, eye data also reflected the reward contingencies of the task as has been shown previously. The monkeys had larger pupil dilation in response to the high reward (HH) condition (see Supplementary Fig. 1*B*); this is thought to be due to increased arousal in response to reward (Varazzani et al. 2015). Moreover, in both monkeys the rate of MSs was reduced during high-reward conditions, which may facilitate the detection of stimuli (Lowet et al. 2018). In one monkey this rate reduction was significant (see Supplementary Fig. 1*C*; chi-squared test; *P* = 0.057 and *P* = 0.012 for monkeys H and K, respectively). These behavioral results suggest that the monkeys were able to change their behavior to increase reward and that both pupil size and MS rate reflected the reward value of trials.

### Reward Cues Outside the Receptive Field Suppress Neural Activity

**Figure 2:**
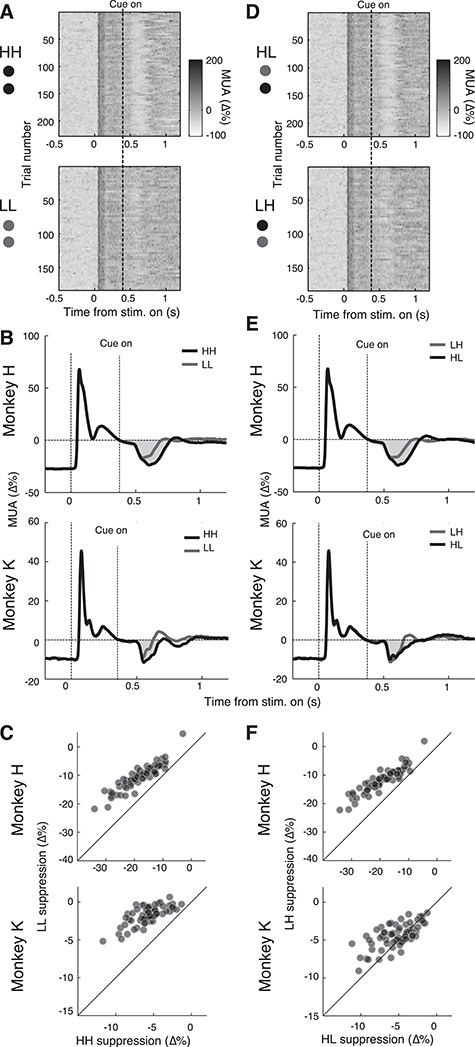
Neural activity is suppressed in response to reward cue onset. (*A* and *D*) MUA responses for all completed trials from one session and electrode (session 1, electrode 29) from monkey H for all reward conditions. The *z*-axis was truncated at 200% for display purposes. (*B* and *E*) Cue response grand average across electrodes. Suppression below stimulus baseline highlighted in gray. (*B*) High reward (condition HH) causes more suppression than low reward (condition LL). (*E*) Only monkey H shows more suppression for HL than LH. (*C* and *F*) Scatter plots of the suppression per electrode. (*C*) High reward (condition HH) causes significantly more suppression than low reward (condition LL) in both monkeys across channels (one-sided WRS, *n* = 63).

We next investigated if reward had any effect on neural activity in visual area V4 using a chronically implanted Utah array (see Methods). In the initial phase of the task, the precue period, increased MUA was observed across all electrodes when a grating stimulus was placed near the RF centers of the electrode array. We then cued reward value by changing the color of the saccade targets placed in the RF surround, between 5 and 11° (visual degrees) away from the RF centers of the recorded neurons. This resulted in a strong suppression approximately 100 ms after the onset of the reward cues (see Fig. 2*A*). Using a one-sided Wilcoxon rank sum (WRS) test, we found that responses were significantly suppressed across recording sites compared to the precue period (*n* = 63, *P* < 0.001 in monkeys H and K). This suppression of stimulus activity was so strong that at some recording sites, the activity decreased below the level of the prestimulus activity. The minimum across the recording sites was −20.85 ± 0.74% and −10.81 ± 0.43% of the precue baseline activity in monkeys H and K, respectively. We interpret this suppression of MUA responses to the grating stimulus as a consequence of the reward cues drawing attention away from it.

**Figure 3:**
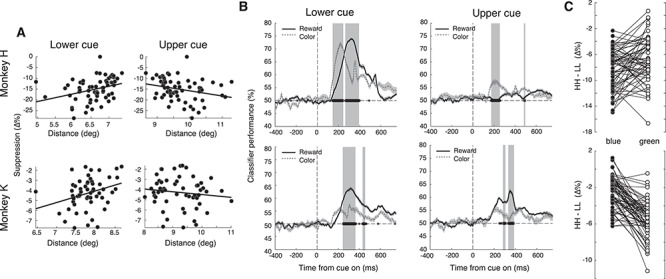
Neural response to the reward cue is due to surround suppression and is influenced by both reward value and color. (*A*) Scatter plot of the strength of MUA suppression with increasing distance of RF centers from the upper and lower cue. Lines are regressions fit separately to each condition. For the lower cue, the regression had positive slopes (of 3.54 and 1.15 in monkeys H and K, respectively) and was significant in both monkeys (*P* = 0.011 and *P* = 0.023, respectively). Note that in monkey K there was a greater distance of the receptive fields from the lower cue (see also Fig. 1*C*). (*B*) Performance of classifier on reward (black lines) and color (dotted lines). Gray shading marks a significant (*P* < 0.01, Holm–Bonferroni corrected) difference between classification performances. Error bars indicate SEM. Note that in monkey H classification performance is poor for the upper cue. (*C*) Difference between HH and LL conditions per electrode split by cue color. Sloped lines indicate an interaction between the suppressive drive from color and reward.

The suppressive effects of the reward cues were strong in all conditions but differed between the reward conditions of our task (see Supplementary Tables 1 and 2). The difference in suppression between HH and LL can be clearly seen in every trial in responses recorded from an example electrode (Fig. 2*A*), and this pattern held over all sessions and electrodes for both monkeys (see Fig. 2*B*). We found that there was stronger suppression after the onset of high reward-predicting cues in the HH condition (minimum of −24.74 ± 0.89% and −12.45 ± 0.49% for monkeys H and K, respectively) than after low reward in the LL condition (minimum of −18.33 ± 0.64% and −9.85 ± 0.39% for monkeys H and K, respectively). Thus the difference in suppression between high versus low reward-predicting conditions was 6.42% and 2.60% in monkeys H and K, respectively. To ensure that the suppression was not due to the MS rate changes that we found behaviorally, we excluded all trials which had detectable MSs in the 600-ms interval following the cue. While this led to the elimination of about half of the trials, the suppression continued to be stronger in the HH than in the LL condition (see Supplementary Fig. 3). To test if the increased suppression with high reward was present across the population, on each trial we subtracted the average activity of the 0.2 s immediately following suppression from the average activity of 0.2 s period of sustained firing just before it (see Methods). We found that in both monkeys, there was a significantly larger suppression in the HH compared to the LL condition across all recording sites (WRS; *P* < 0.001, *n* = 63, see Fig. 3*C*).

We hypothesized that it was the reward value of the visual cue that causes the suppression, but it is also conceivable that it was the reward value of the trial itself. To distinguish between these possibilities, we used the variable reward conditions HL and LH, as they signaled equal expected rewards for a trial, but the locations of the high and low reward cues were different. In monkey H the suppression caused by condition HL was very similar to that caused by HH, while condition LH was similar to LL (see Fig. 2*D*–*F*). Therefore, it was not the expected reward of the trial that determined the amount of suppression. Rather what mattered was the reward value predicted by the lower cue which, when compared to the upper cue, happened to be nearer the RF centers of the recorded neurons in this monkey (see Fig. 1*C*). Thus, we conjectured that the observed suppression was caused by the reward value of the cue that was in the suppressive surround of the neurons (Reynolds and Heeger 2009). We found significantly greater suppression in both monkeys when the neurons’ RFs were closer to the lower cue (see Fig. 3*A*; linear regression; *P* = 0.011 and *P* = 0.023 for monkeys H and K, respectively). For further details of the suppressive surround of the arrays, refer to Kienitz et al. (2018). Additionally, Fig. 3*B* shows that for monkey H, a simple naive Bayes classifier could distinguish between task conditions at well above chance level for the lower cue but much less so for the upper cue. For the lower cue, the peak classification of reward was 73.9 ± 0.5%, color peak classification 72.0 ± 0.9%, and for the upper cue reward was only 54.0 ± 0.6%, color peak classification 57.2 ± 0.9%, indicating that it was the lower cue causing the majority of the suppression effect. Overall, during this reward cueing period, we found evidence for a change in suppressive drive of the reward cues with increased reward association.

### Stimulus Color and Reward Interact in Causing Neural Suppression

As area V4 is a color-sensitive area (Zeki 1973; Roe et al. 2012; Li et al. 2014), we wanted to ensure that the observed change in suppression was due to the reward value of the cue and not due to its color or luminance. We therefore switched the reward value of the colors during the second week of recordings (see Methods). Color did indeed have an effect on the strength of suppression. In both monkeys blue cues caused more suppression than green cues. However, the reward-induced suppression effect persisted even when color was taken into account. In both monkeys the effect of reward and color were nonadditive, as an interaction term of a linear model was found to be significant with *P* < 0.001 and *P* < 0.001 for monkeys H and K, respectively (see Supplementary Tables 1 and 2). This interaction can be visualized in Figure 3*C* by the sloped line observed for most recording sites between the green and blue conditions’ change in reward suppression. This interaction was expected as the normalization mechanism of attention predicts that attention (in this case reward) multiples the stimulus drive (in this case color) producing a nonadditive interaction between the two factors.

To further investigate the interaction of color and reward on the observed suppression, we trained a classifier to identify either the reward value (H or L) or the color (blue or green) of the lower cue or the upper cue based on the recorded neural activity (see Methods). The classifier performed similarly well for both reward and color (see Fig. 3*B*; gray shading shows significant differences with *P* < 0.01). In monkey K the reward information contained in the suppressed postcue responses was significantly stronger than the color information (see Fig. 3*B* bottom panels, for the lower cue, the peak classification of reward was 63.8 ± 0.7%, color peak classification 57.2 ± 0.9%). In monkey H the early postcue responses (~200 ms after cue onset) contained more information about the color (peak classification 72.0 ± 0.9%), but later response components (~350 ms after cue onset) contained more information about the reward (peak classification of 73.9 ± 0.5%). This was the case only for the lower cue; the upper cue was difficult to classify for monkey H as described in the previous section. In summary, we found that both the reward and color of the cues interact nonadditively and with individual timings to cause the observed neural suppression.

### Neural Activity Does Not Reflect Feature Attention During Perceptual Decision-Making

**Figure 4:**
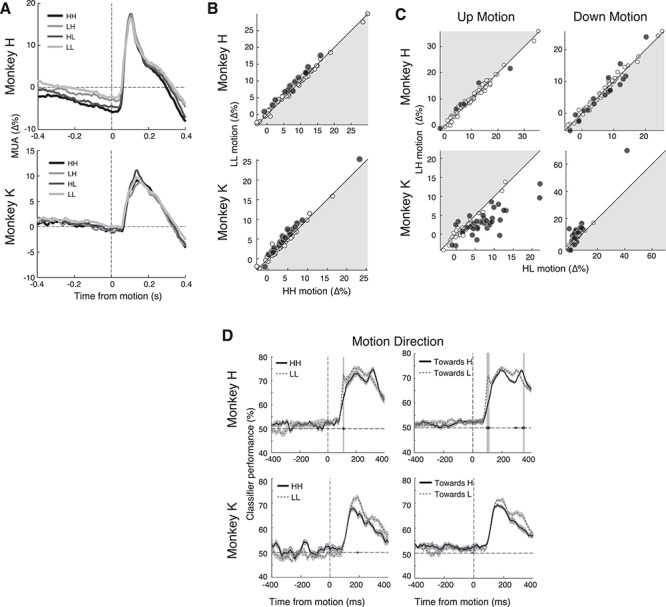
Neural responses to motion direction are not stronger when the expected reward is higher. (*A*) Motion response MUA grand average across electrodes. There is little difference in the motion response between conditions, but a sustained suppression in condition HH and HL is visible in monkey H before motion onset. (*B*) Scatter of neural responses to motion in HH and LL condition. Note that high reward does not cause any overall increase in responses (points would be located in the gray-shaded area). Black points are significant (WRS). (*C*) Scatter plots of neural responses to upward and downward motion per electrode. Black points are significant (WRS). There is no increase in response to downward motion in HL or increase in response to upward motion in LH conditions (points would be located in the gray-shaded area). (*D*) Performance of classifier on motion direction for different conditions. Gray shading marks a significant (*P* < 0.01, Holm–Bonferroni corrected) difference between classification performances. Error bars indicate SEM. Note that, contrary to expectations, most time points that show significant differences in monkey H have an increased classification performance for low reward.

According to previous evidence of goal-driven feature attention (Cohen and Maunsell 2011), one might have expected that the highly rewarded motion direction would be associated with an increase of the neurons’ responses. This is, however, not what we found (see Fig. 4*A*). Instead, after the initial reward cue-driven suppression, neural activity returned to the precue baseline level in monkey H (see Fig. 2) and remained slightly suppressed in monkey K (see Supplementary Fig. 4). There was no significant increase of the population motion response to HH as compared to LL trials for either monkey (see Fig. 4*B*; one-sided WRS; monkey H, *P* = 0.717, and monkey K, *P* = 0.360). Likewise, in the unevenly rewarded trials (i.e., LH and HL), there was no significant increase in the population motion response associated with higher reward (see Fig. 4*C*; one-sided WRS; during condition LH upward, *P* = 0.540 and *P* = 1.00, and during HL downward motion, *P* = 0.543 and *P* = 0.708, for monkeys H and K, respectively). As expected (Ferrera et al. 1994; Tolias et al. 2005), a subset of recording sites displayed a preference for motion direction that was independent of attention or reward-associated response modulation. In monkeys H and K, significant differences in the neurons’ upward versus downward motion direction preference were found in 92.1% (58 of 63) and 82.5% (52 of 63) of sites in monkeys H and K, respectively (see Supplementary Fig. 5*A*). Although we found that the population motion responses to highly rewarded directions did not increase compared to lower rewarded directions, it is still possible for these responses to be more informative about the direction of stimulus motion, for example, through a decrease in variability. However, analysis with classifiers provided no evidence for better decoding of motion direction associated with a higher reward compared to motion associated with a lower reward (see Fig. 4*D*). We therefore did not find any neural evidence of goal-driven attention to the more highly rewarded motion feature in this task.

## Discussion

In this study, we found evidence for value-driven attentional mechanisms in area V4 of the visual association cortex. When reward-predicting visual cues appeared in the RF surrounds of the assessed neurons, the neural responses to the target stimulus positioned in the RF were strongly suppressed. The magnitude of this suppression depended both on the reward amount and the color of the cue, indicating interactions between reward and visual information. In previous research, attention has been described to contribute to the normalization of stimulus information in the visual cortex (Lee and Maunsell 2009; Reynolds and Heeger 2009). Our findings suggest that reward-predicting cues engage these attentional normalization mechanisms as predicted by the value-driven attention theory (Anderson 2016).

Our results cannot be accounted for by classical theories of attention alone (Moran and Desimone 1985; Kastner and Ungerleider 2000). A goal-driven attention theory would have predicted an increase in responses to the target stimulus during trials associated with high reward (Baruni et al. 2015), which we did not observe. Alternatively, responses to the target stimulus could have been characterized by enhanced signal to noise ratios (Verghese 2001), which we did not observe either. On the contrary, we observed slightly less information about stimulus direction during trials associated with high reward (see Fig. 4). The value-driven attention theory can account for our findings. It posits that reward-associated stimuli assume increased attentional priority, even if this attentional capture is disadvantageous in the current task (Anderson and Yantis 2013). In our experiments, reward cues, which were not necessary for successful task performance, captured attention and through attentional normalization mechanisms caused suppression of responses to the task-relevant (target) stimulus. This evidence for a value-driven attentional mechanism in area V4 agrees with previous psychophysical experiments (Hickey and Van Zoest 2012; Anderson and Yantis 2013). It is also consistent with electrophysiological results from other visual areas of nonhuman primates showing that reward and attention cause similar effects (Peck et al. 2009; Stanisor et al. 2013).

### Compatibility with Previous Work and Current Models of Attention

Our results are well described by combining the normalization model of attention (Lee and Maunsell 2009; Reynolds and Heeger 2009) with the value-driven attention concept (Anderson 2013). This extended framework allows us to make a number of predictions and to compare these with the results of previous studies. Baruni et al. (2015) found that increased absolute reward caused similar changes in firing rates, correlations, and local field potential (LFP) power as selective attention. However, they did not find further firing rate changes caused by relative reward differences. As mentioned in the introduction, this is likely due to the fact that their stimuli were too far outside the RF surround to engage normalization mechanisms (see Introduction for further details). Therefore the findings of Baruni et al. are compatible with and can be accounted for by our framework. In a study by Luo and Maunsell (2015), two gratings were presented, and monkeys had to perform a change detection task on one of them. These authors found that the responses of neurons were stronger for the grating predicting higher reward when the relative award associated with the two gratings differed. However, when the relative award was associated with the decision, the responses of the neurons did not follow the same pattern. When one grating had more reward for a correct detection while the other had more reward for a correct rejection, neural responses to the two gratings were similar. Thus, neural responses changed as a function of the reward predicted by the stimulus rather than that associated with the execution of a particular response, which again aligns with our framework. Sundberg et al. (2009) found that responses to an unattended center stimulus were more strongly suppressed by an attended stimulus presented in the surround RF than by another unattended stimulus. Their results were very similar to ours, even though in their task, unlike ours, reward and behavioral relevance were coupled. Shifts in attention caused maximal response suppression 210 ms poststimulus and thus with the same latency as our reward-predicting stimuli (~200 ms poststimulus). Feng et al. (2009) hypothesized that a value-maximization process for the selection of rewarded options might be performed in the lateral intraparietal cortex (LIP). A mechanism like value-driven attention in lower areas could help to ensure that the representation of reward-related cues necessary to perform such a calculation are passed on for further processing in higher areas. In conclusion, the framework of value-driven attention accounts well for previously reported attention effects in area V4. Because attention effects are very sensitive to experimental conditions (Sundberg et al. 2009), seemingly different outcomes can be explained by differences in experimental paradigms.

### Biological Mechanisms of Reward-Related Neural Suppression

While our framework can accommodate our own and others’ findings in area V4, it does not suggest a biological mechanism for our finding of increased suppression with reward. Area V4 has previously been described as a color processing region (Zeki 1973; Roe et al. 2012; Li et al. 2014) and therefore might be expected to preferentially represent information about color. However, our classification analysis indicated that V4 suppressive responses contain equal or less information about color identity than about reward value. Additionally, the early and late segments of the suppressive responses contained preferentially color and reward information, respectively. We also found that for most recorded sites, suppression due to color and reward was not additive. Rather, color and reward interacted significantly. Taken together this suggests that the suppression resulted from two different but interacting mechanisms. The early suppression is most likely due to the classical feature selective surround inhibition that has short latency and is mediated mainly by horizontal (tangential) connections within V4 (Ungerleider et al. 2008). The later suppression, in contrast, is caused by the reward dependent capture of attention that has a longer latency and is with all likelihood mediated by top-down projections (Ungerleider et al. 2008). However, an alternative possibility is that the later reward-related suppression is not caused by top-down projections but is instead caused by dopaminergic inputs. This was suggested by Arsenault et al. (2013), who reported decreased fMRI activity to reward without a visual stimulus in the visual cortex (including visual area V4). This negative response, which has been linked to suppression (Shmuel et al. 2006), was reduced by a dopamine receptor antagonist. These authors hypothesized that this decreased fMRI activity with reward in area V4 may have been caused by activation of dopaminergic receptors in feedforward or feedback areas (Noudoost and Moore 2011) or by direct dopaminergic connections to area V4, which could also be the case in our findings. In support of the likely role of dopamine in our experiment, human positron emission tomography studies have observed a presumed increase in dopamine release to a task-irrelevant distractor which correlated with the extent of its attentional capture (Anderson et al. 2016, 2017). Experiments designed specifically to distinguish between these possibilities are necessary to elucidate the biological mechanism of the value-driven suppression observed in our study.

To add further support for the framework of value-driven attention, our experimental design could be extended in multiple ways. In our experiments we were not able to record from the neurons representing the reward cue simultaneously with neurons representing the discriminada. Our prediction for such a scenario is that at the same time as we observe suppression in the response to the discriminada, we should see an increased response to a high reward cue. These responses should be correlated trial to trial, because we predict that greater reward cue responses should lead to stronger suppression via normalization mechanisms. Another important variation would be to determine the strength of suppression as a function of the distance between the reward cue and the discriminandum. We predict a decay of suppression with distance. This could be compared to the pattern of suppression caused by classical surround suppression without reward and would allow a full attention normalization model to be fit to the data. Additionally, by observing the time course of suppression at different cues to discriminandum distances, it may be possible to determine the mechanism of the reward-related suppression (e.g., mediated by horizontal or top-down connections). Finally, it would be useful to use a variation of the task with either more stimuli or the additional possibility of no motion so that sensitivity measures (like d’) could be used to prove that selective attention was engaged by the monkey.

In conclusion, our present results, together with previous observations, support a framework of value-driven attention. This raises new questions about the neural mechanisms mediating the interaction between reward and attention systems. Further studies will need to clarify to which extent the various forms of attentional control, spatial, feature selective, or goal-oriented attention are modulated by reward.

## Supplementary Material

rewardV4Shapcott_SI_final_bhaa079
