# Supplementary material for: Reward-Related Suppression of Neural Activity in Macaque Visual Area V4
Source: Cereb Cortex. 2020 Apr 30;30(9):4871–81. doi: 10.1093/cercor/bhaa079 (PMC7391271; doi:10.1093/cercor/bhaa079)
Supplement: rewardV4Shapcott_SI_final_bhaa079 [file rewardv4shapcott_si_final_bhaa079.pdf]

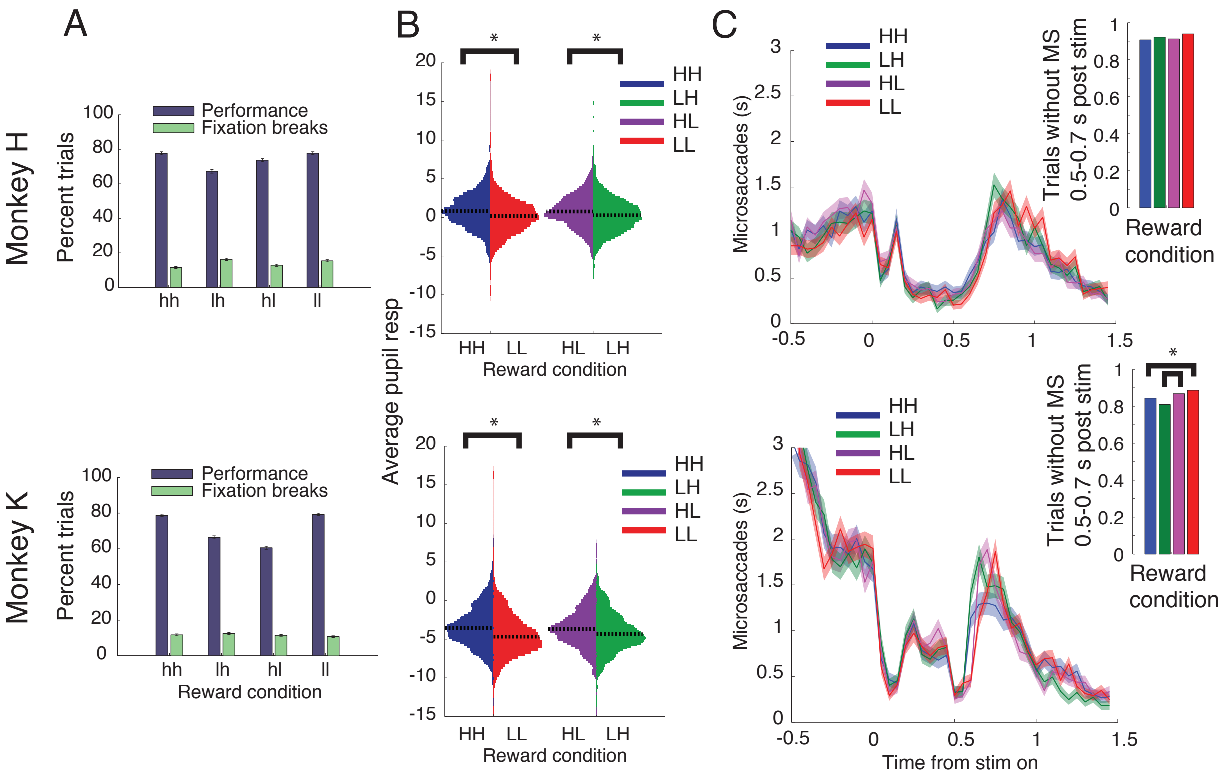

Fig. S1. Additional behavioral results
A. Overall task performance. Performance percentage was calculated from only the completed trials. Performance is best for HH and LL as there is less bias in these conditions. Error bars indicate SEM. B. Average pupil responses after reward cue onset. When there is low expected reward (LL) the pupil size is smaller than when there is high expected reward (HH) (0.63 % and 1.11 % in monkey H and K respectively, Wilcoxon rank-sum test (WRS) $p<0.001$ for both monkeys) and intermediate reward (HL and LH) is less than high reward (WRS $p<0.01$ for both monkeys). Unexpectedly, in both monkeys there is a separation between HL and LH conditions, despite the expected reward being the same, (WRS $p<0.001$ for both monkeys). However, the magnitude of this difference was less than that between HH and LL (0.49 % and 0.63 % in monkey H and K respectively). Color indicates reward condition. Histograms were smoothed with a ksdensity kernel, see Methods. C. Microsaccade rate. Microsaccades were modulated throughout the task and differently by the reward conditions. In both monkeys, condition HH had less microsaccades than LL. In all figures * indicates significance.

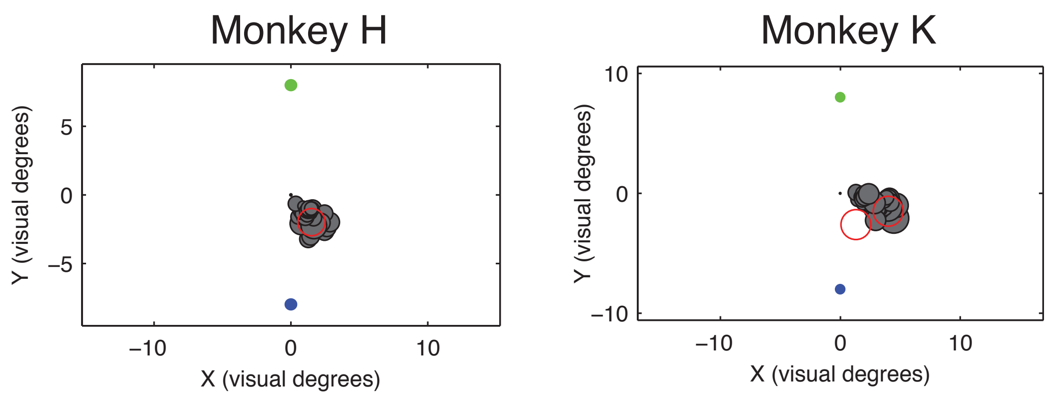

Fig. S2. Receptive field sizes and locations relative to visual display.
Image borders represent the edges of the display screen, presented to scale in units of visual degrees. Central black point at (0,0) is the fixation spot, blue and green dots at (0,-8) and (0,8) respectively are the reward cues. Grey circles represent the location and sizes of the central receptive fields (see Methods) from 63 electrodes. Red circles indicate the size and placement of the target grating stimulus for each monkey. For monkey H the target was positioned within the majority of V4 RFs. For monkey K the target was positioned to evoke a response in the RFs but was not centered on the RFs (leftmost red circle, 88 % of trials) in order to evoke responses from a second array of electrodes in area V1 (data not shown). In the remaining 12 % of trials the grating was positioned within the majority of V4 RFs (as for monkey H). Data from both positions was pooled for the analysis.

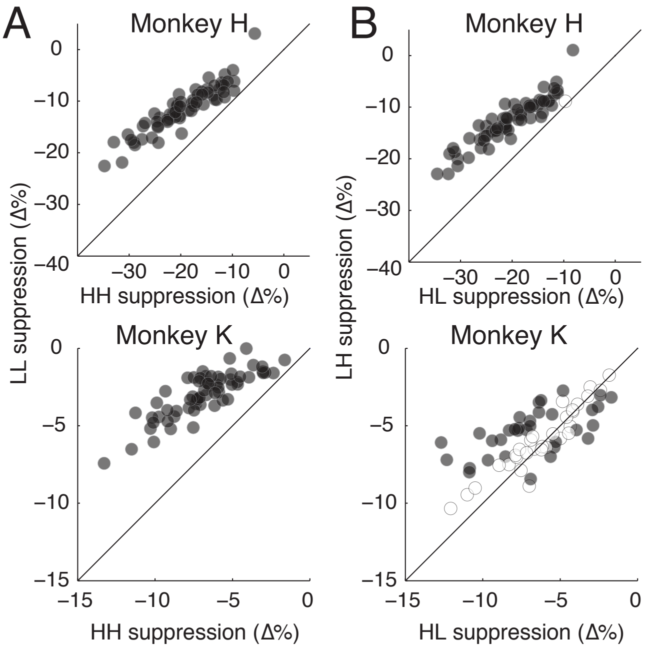

Fig. S3. MUA suppression for trials without MS
Scatter plots of the suppression per electrode, results look qualitatively similar to Figure 2 C and F, which include trials with MS. Black points are significant (WRS). A. High reward (condition HH) causes more suppression than low reward (condition LL). B. In monkey H HL causes more suppression than LH but that is not the case in monkey K.

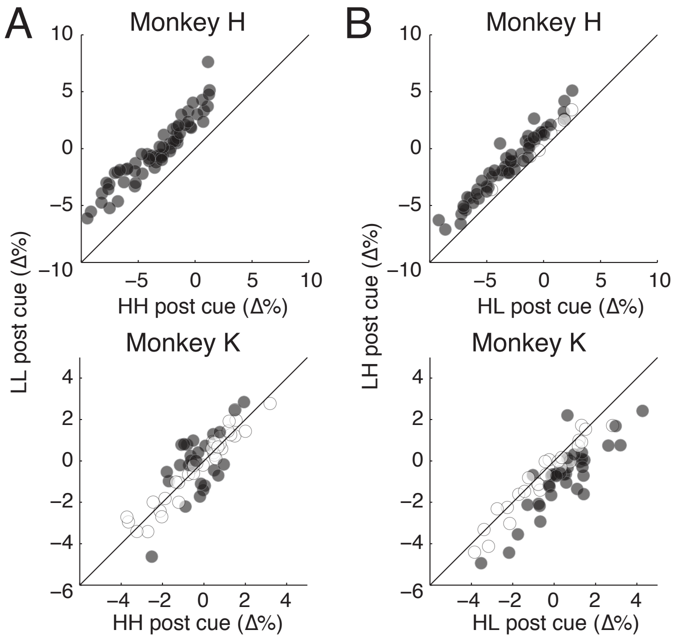

Fig. S4. Late suppression of activity
A and B. Scatter plots of the median suppression per electrode 0.6 to 1 s post cue. Note that only high reward conditions in monkey H still remain more suppressed at this time period. Black points are significant (WRS). A. Condition HH vs LL. In monkey H 100% of recording sites (63 of 63) are significantly suppressed, while in monkey K only 60.3 % of recording sites were still more suppressed and 28.6 % (18 of 63 electrodes) were significantly more suppressed. B. Condition HL vs LH.

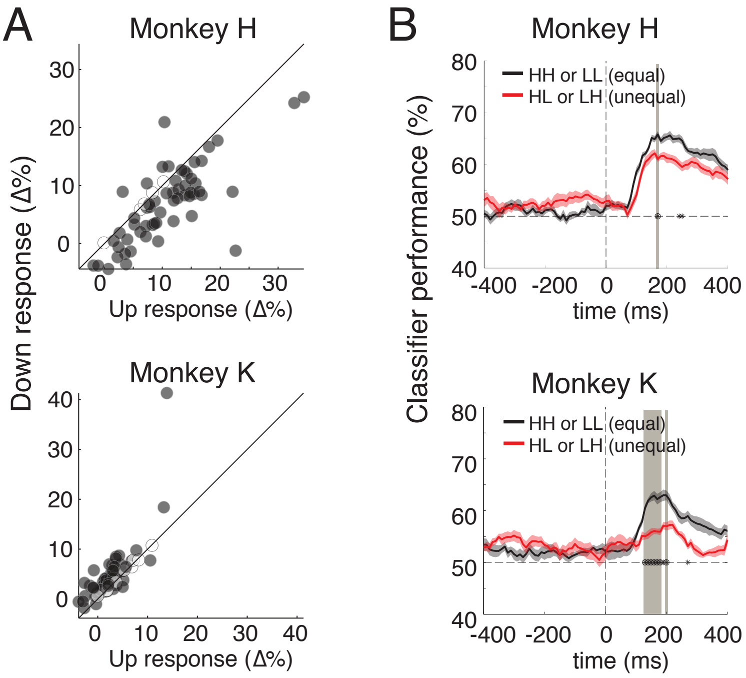

Fig. S5. Additional MUA responses to motion and classification of choice
A. Scatter plots of neural responses to upwards vs downwards motion across all conditions. Black points are significant (WRS). B. Performance of classifier on choice of saccade direction from motion responses for equal reward conditions (HH and LL) and for unequal reward conditions (HL and LH). Note that the classification of choice is better in equal reward conditions, which indicates that in unequally rewarded conditions motion is less relevant to behavioral choice. Grey shading marks a significant ($p<0.01$, Holm-Bonferroni corrected) difference between classification performances.

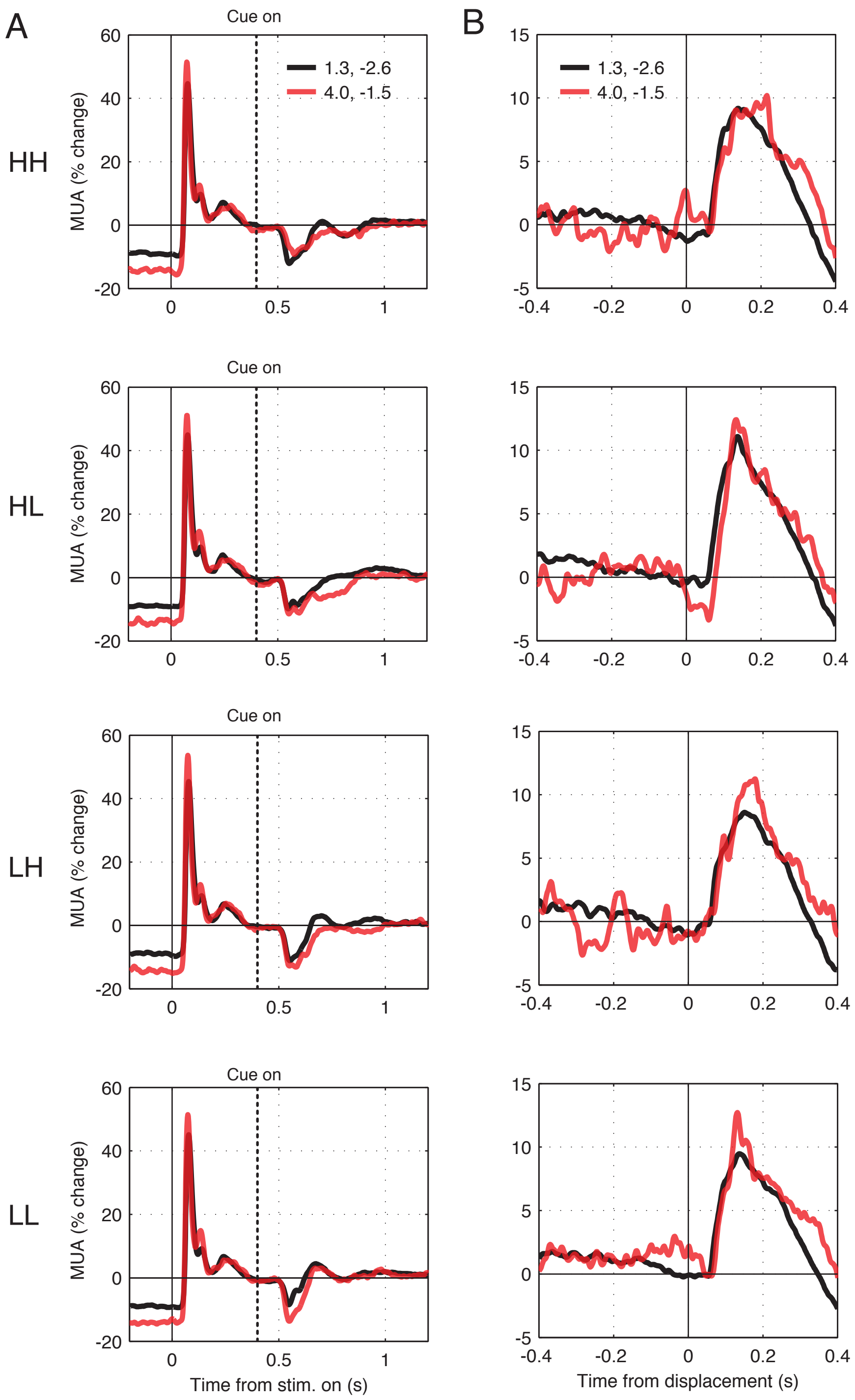

Fig. S6. Responses for Monkey K split by stimulus position

A. Responses to the stimulus onset for both stimulus positions for each reward condition. Colors signify the 2 stimulus positions. While the stimulus closer to the RFs does cause stronger responses, because the baseline is to the sustained activity the difference can mainly be observed in the pre stimulus onset period. Differences in the cue response are likely due to the color B. Responses to the motion onset for both stimulus positions for each reward condition. Colors signify the 2 stimulus positions. Again, due to baseling to the sustained activity little difference can be seen in the two stimulus positions.

	SumSq	DF	MeanSq	F	pValue
**reward**	5.7331e+06	3	1.911e+06	8642	0
**colour**	1.2518e+05	1	1.2518e+05	566.08	4.8503e-125
**channel**	1.107e+07	62	1.7855e+05	807.44	0
**repetition**	9.842e+05	4	2.4605e+05	1112.7	0
**reward:colour**	5.2749e+06	3	1.7583e+06	7951.3	0
**Error**	9.1249e+07	4.1264e+05	221.13		

Table S1

ANOVA results for Monkey H. Linear regression with model:

*suppression ~ 1 + reward + color + channel + repetition + reward*color*

	SumSq	DF	MeanSq	F	pValue
**reward**	1.2236e+06	3	4.0788e+05	2497.1	0
**colour**	1.2373e+05	1	1.2373e+05	757.51	1.3225e-166
**channel**	1.0506e+06	62	16945	103.74	0
**stim_position**	32841	1	32841	201.06	1.2597e-45
**repetition**	10235	4	2558.7	15.665	8.0261e-13
**reward:colour**	22896	3	7632.1	46.725	3.5079e-30
**Error**	6.7638e+07	4.1409e+05	163.34		

Table S2

ANOVA results for Monkey K. Linear regression with model:

*suppression ~ 1 + reward + color + channel + stim_position + repetition + reward*color*
